# Clinical Significance of Initial and Converted Cardiac Rhythms in Extracorporeal Cardiopulmonary Resuscitation for Patients with Refractory Out-of-Hospital Cardiac Arrest: A Nationwide Observational Study

**DOI:** 10.3390/jcm14145066

**Published:** 2025-07-17

**Authors:** Sola Kim, Jae-Guk Kim, Gu-Hyun Kang, Yong-Soo Jang, Wonhee Kim, Hyun-Young Choi, Chiwon Ahn

**Affiliations:** 1 Department of Emergency Medicine, Dongtan Sacred Heart Hospital, Hallym University College of Medicine, Keunjaebong-gil, Hwaseong-si 18450, Republic of Korea; solarsolakim@hallym.or.kr; 2 Department of Emergency Medicine, Kangnam Sacred Heart Hospital, Hallym University College of Medicine, Seoul 07441, Republic of Korea; emkang@hallym.or.kr (G.-H.K.); amicoys@hallym.or.kr (Y.-S.J.); wonsee02@hallym.or.kr (W.K.); chy6049@hallym.or.kr (H.-Y.C.); 3 Department of Emergency Medicine, College of Medicine, Chung-Ang University, Seoul 06974, Republic of Korea; cahn@cau.ac.kr

**Keywords:** extracorporeal cardiopulmonary resuscitation, outcome, out-of-hospital cardiac arrest, heart rhythm, rhythm conversion

## Abstract

**Background/Objectives:** Initial cardiac rhythm is a known prognostic indicator in out-of-hospital cardiac arrest (OHCA). However, the impact of rhythm conversion during cardiopulmonary resuscitation (CPR) on outcomes in patients undergoing extracorporeal CPR (ECPR) remains unclear. This study evaluated the association between initial and converted cardiac rhythms and outcomes in patients with refractory OHCA treated with ECPR. **Methods**: This nationwide retrospective observational study analyzed data from the Out-of-Hospital Cardiac Arrest Surveillance registry in South Korea (2008–2022). Patients were categorized into three groups: initial shockable rhythm (SR), non-shockable rhythm (NSR) converted to SR, and refractory NSR. The primary outcome was survival to hospital discharge; the secondary outcome was favorable neurological status (CPC 1–2). **Results**: Among 681 patients, 161 had initial SR, 345 had converted SR, and 175 had refractory NSR. Before matching, survival and CPC 1–2 rates were highest in the initial SR group (21.1% and 15.5%), followed by the converted SR group (19.4% and 11.6%), and lowest in the refractory NSR group (9.7% and 4.0%) (*p* < 0.01). After matching, CPC 1–2 remained significantly higher in the initial SR group (14.4%) and in the converted SR group (9.3%) vs. the refractory NSR group (5.1%, *p* = 0.016; 3.7%, *p* = 0.042). Persistent NSR was independently associated with poor neurological outcomes compared to both initial SR (AOR 0.337, *p* = 0.037) and converted SR (AOR 0.283, *p* = 0.020). **Conclusions**: Rhythm conversion from NSR to SR before ECPR was associated with significantly improved neurological outcomes. Rhythm conversion may serve as a prognostic marker and resuscitation target to guide ECPR decisions.

## 1. Introduction

Out-of-hospital cardiac arrest (OHCA) remains a major global public health challenge, with survival rates continuing to be dismally low despite advances in cardiopulmonary resuscitation (CPR) and post-resuscitation care [1]. Although the incidence of OHCA varies across regions, the overall prognosis remains poor, with only a small proportion of patients achieving meaningful neurological recovery [2]. Conventional resuscitative interventions such as high-quality chest compressions, defibrillation, and advanced airway management have contributed to incremental improvements in outcomes [3]. However, these measures are often insufficient in cases of refractory cardiac arrest, necessitating consideration of advanced resuscitation strategies [4].

Extracorporeal cardiopulmonary resuscitation (ECPR) has emerged as a promising therapeutic option for selected patients with refractory OHCA. ECPR involves the rapid initiation of veno-arterial extracorporeal membrane oxygenation during active resuscitation, providing temporary circulatory and oxygenation support when spontaneous circulation cannot be restored by conventional methods [4,5]. Observational studies suggest that ECPR may improve survival and neurological outcomes in carefully selected patients. Nonetheless, identifying individuals most likely to benefit from ECPR remains a significant clinical challenge, as outcomes are influenced by multiple factors, including the duration of CPR, the underlying etiology of arrest, and the presence of reversible causes [5].

Among the established prognostic indicators for OHCA, the initial cardiac rhythm has consistently been linked to clinical outcomes. Patients presenting with a shockable rhythm (SR), such as ventricular fibrillation or pulseless ventricular tachycardia, typically have better survival and neurological recovery compared to those with a non-shockable rhythm (NSR), including asystole and pulseless electrical activity [6]. While this association is well-documented, limited evidence exists regarding the prognostic value of rhythm conversion, specifically the transition from NSR to SR during CPR in the context of ECPR [7]. Such a conversion may reflect an evolving physiological state and the potential for recovery, possibly serving as a clinically meaningful predictor of treatment response [8].

Given the established importance of initial cardiac rhythm and the potential prognostic implications of rhythm conversion during resuscitation, further investigation is warranted. This study aims to evaluate the associations between initial cardiac rhythm, rhythm conversion from NSR to SR, and clinical outcomes in patients with refractory OHCA managed with ECPR.

## 2. Materials and Methods

### 2.1. Study Design and Settings

This retrospective observational study utilized data from the Out-of-Hospital Cardiac Arrest Surveillance (OHCAS) database, maintained by the Korean Center for Disease Control and Prevention (KCDC). The study period spanned from January 2008 to December 2022.

The OHCAS registry encompasses all 17 administrative provinces in South Korea, a population of approximately 50 million people. It provides comprehensive and standardized data to support research on OHCA. Ethical approval for this study was obtained from the Institutional Review Board (IRB) of Kangnam Sacred Heart Hospital (IRB No. HKS 2025-02-009). Authorization for data use was granted by the KCDC in 2025. Due to the retrospective design and use of anonymized data, the requirement for informed consent was waived.

This study was conducted in accordance with the Strengthening the Reporting of Observational Studies in Epidemiology guidelines to ensure methodological transparency and reporting consistency.

### 2.2. Data Source

The OHCAS registry is a nationwide, population-based database that collects data on OHCA cases assessed and managed by emergency medical service (EMS) personnel in South Korea. It includes systematically collected clinical, demographic, and outcome-related data derived from prehospital EMS records and hospital-based medical documentation.

The registry is managed by the KCDC, which also oversees data quality assurance. Trained medical record reviewers from the KCDC conduct on-site audits at all emergency departments and hospitals that admit patients with OHCA. These reviewers verify and abstract medical data to ensure completeness and accuracy.

The registry’s design and case-reporting forms were developed based on internationally recognized Utstein-style guidelines and protocols from the Resuscitation Outcomes Consortium Project, aligning the registry with global standards in cardiac arrest research.

### 2.3. Study Population

A total of 423,437 individuals with OHCA were registered in the OHCAS database between January 2008 and December 2022. Patients were excluded if they met any of the following criteria: age < 18 years, cardiac arrest due to non-medical causes (e.g., trauma, drowning, intoxication), sustained return of spontaneous circulation (ROSC) prior to ECPR implementation, death on arrival, documented do-not-resuscitate (DNR) orders, absence of ECPR implementation, unknown initial cardiac rhythm, or missing outcome data.

After applying these criteria, patients who received ECPR were categorized into three groups based on their initial and converted cardiac rhythms before ECPR initiation:

- Initial SR group: Patients who presented with ventricular fibrillation or pulseless ventricular tachycardia.
- Initial NSR converted to SR group: Patients whose initial rhythm was asystole or pulseless electrical activity but who converted to SR during resuscitation.
- Refractory NSR group: Patients who remained in a persistent non-shockable rhythm without conversion.

### 2.4. Variables

The following variables were collected and analyzed: demographic data (age and sex); prehospital factors (witnessed arrest and bystander CPR); initial cardiac rhythm (SR vs. NSR); preexisting comorbidities; and in-hospital post-resuscitation care, including reperfusion therapy and targeted temperature management (TTM).

Preexisting comorbidities were defined as clinically diagnosed conditions documented in the medical records prior to the cardiac arrest, including hypertension, diabetes mellitus, chronic kidney disease, respiratory disease, and dyslipidemia (Table S1). A cardiac cause of arrest was defined as a presumed cardiac etiology, such as ischemic heart disease, arrhythmia, or cardiac tamponade, particularly in cases of unexpected collapse.

Reperfusion therapy included intravenous thrombolysis or percutaneous coronary intervention (PCI). Transient pre-ECPR ROSC was defined as any palpable pulse or measurable blood pressure sustained for more than 1 min but less than 20 min prior to ECPR cannulation, either before or after hospital arrival [9].

The application and modality of TTM were determined by the attending physicians in accordance with hospital protocols. Cooling methods included surface or intravascular systems with automated temperature feedback mechanisms, such as Arctic Sun^®^ (Medivance Corp., Louisville, KY, USA) and CoolGard 3000^®^ (Alsius Corp., Irvine, CA, USA). TTM protocols adhered to the American Heart Association guidelines and targeted a temperature range of 32–36 °C for a maintenance duration of 12–24 h [10,11].

The time interval from EMS call or witnessed arrest to ED arrival was defined as the duration from EMS activation or witnessed cardiac arrest to ED presentation. The time interval from ED arrival to ECPR initiation was defined as the duration from ED arrival to the initiation of ECPR.

The presence of a shockable rhythm or conversion to a shockable rhythm was determined based on electrocardiogram (EKG) recordings or by the administration of defibrillation by EMS personnel or emergency physicians, either in the prehospital setting or upon ED arrival. Neurological outcomes were assessed using the Cerebral Performance Category (CPC) scale, with CPC scores of 1–2 indicating favorable neurological status and scores of 3–5 indicating unfavorable outcomes (Table S2).

### 2.5. Outcome Measure

The primary outcome was survival to hospital discharge. The secondary outcome was a favorable neurological outcome, defined as a CPC score of 1–2 at hospital discharge.

### 2.6. Statistical Analysis

Descriptive statistics were used to summarize patient characteristics and clinical variables. Continuous variables were presented as medians with interquartile ranges (IQRs), while categorical variables were presented as frequencies and percentages. The normality of continuous variables was assessed using the Kolmogorov–Smirnov test. Categorical variables were compared using Pearson’s chi-square test or Fisher’s exact test, as appropriate. Continuous variables were compared across more than two groups using the Kruskal–Wallis test.

To minimize potential confounding and selection bias, propensity score matching was conducted. A 1:1 nearest-neighbor matching algorithm was applied using a caliper width of 0.2 standard deviations of the logit of the propensity score. Covariates included in the propensity score model were age, sex, bystander CPR, witnessed arrest, presumed cardiac etiology, preexisting comorbidities, use of mechanical CPR, reperfusion therapy, TTM, and transient ROSC prior to ECPR initiation.

The balance between matched groups was assessed using standardized mean differences (SMDs), with an SMD < 0.1 considered indicative of a negligible difference.

For multivariable analysis, variables with a *p*-value < 0.05 in univariate comparisons were included in logistic regression models. Multivariable logistic regression was performed to estimate the adjusted odds ratios (AORs) and 95% confidence intervals (CIs) for survival and favorable neurological outcomes (CPC scores of 1–2). A stepwise backward elimination method was employed to refine the model by sequentially removing non-significant covariates.

All statistical analyses were performed using SPSS software (version 26.0; IBM Corp., Armonk, NY, USA) and R software (version 3.3.2; R Foundation for Statistical Computing, Vienna, Austria). Statistical significance was defined as a two-tailed *p*-value < 0.05.

## 3. Results

### 3.1. Patient Characteristics

**Figure 1:**
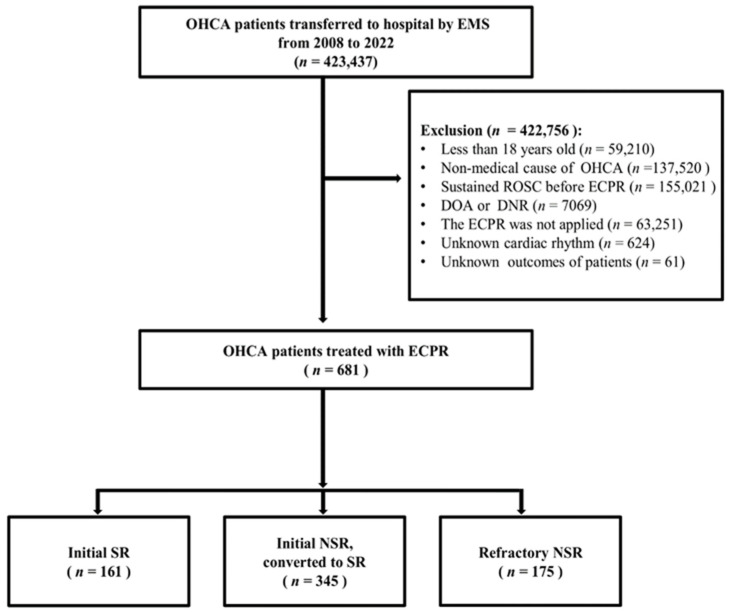
Flowchart of patient inclusions and exclusions in this study. EMS, emergency medical system; OHCA, out-of-hospital cardiac arrest; ROSC, return of spontaneous circulation; ED, emergency department; ECPR, extracorporeal cardiopulmonary resuscitation; TTM, targeted temperature management; DOA, dead on arrival; DNR, do not resuscitate; SR, shockable rhythm; NSR, non-shockable rhythm.

Between January 2008 and December 2022, 423,437 adult patients with OHCA were transported to hospitals across South Korea. Of these, 681 patients who received ECPR were included in the final analysis (Figure 1). These patients were categorized into three groups based on their initial and converted cardiac rhythms prior to ECPR initiation: initial SR group (n = 161), initial NSR converted to SR (converted group, n = 345), and persistent NSR (refractory NSR group, n = 175). Baseline characteristics and clinical outcomes of the three groups are presented in Table 1.

### 3.2. Survival and Neurological Outcome at Hospital Discharge Before Propensity Score Matching

The overall survival rate at discharge was 17.3%. Patients in the initial SR group had the highest survival rate (21.1%), followed by those in the converted rhythm group (19.4%) and the NSR group (9.7%, *p* = 0.008). The overall rate of favorable neurological outcome, defined as a CPC score of 1–2 at discharge, was 10.6%. This outcome was most frequent in the initial SR group (15.5%), followed by the converted rhythm group (11.6%) and the NSR group (4.0%, *p* = 0.002) (Table 1). These findings underscore the prognostic significance of the initial cardiac rhythm and rhythm conversion during CPR in patients undergoing ECPR. Specifically, patients with initial SR and those who converted to SR demonstrated higher survival rates and better neurological outcomes than those with refractory NSR.

### 3.3. Propensity Score Matching for Outcomes at Hospital Discharge Between the Three Groups

To investigate the impact of initial and converted cardiac rhythms on survival and good neurological outcomes (CPC 1–2) in patients treated with ECPR, propensity score matching was performed. Matched analyses compared survival outcomes between patients with initial SR, those with initial NSR converted to SR, and those with refractory NSR. Changes in absolute SMD and dot plots of absolute SMD among patients undergoing ECPR are shown in Figures S1–S3. Outcome comparisons for the matched groups are summarized below.

**Figure 2:**
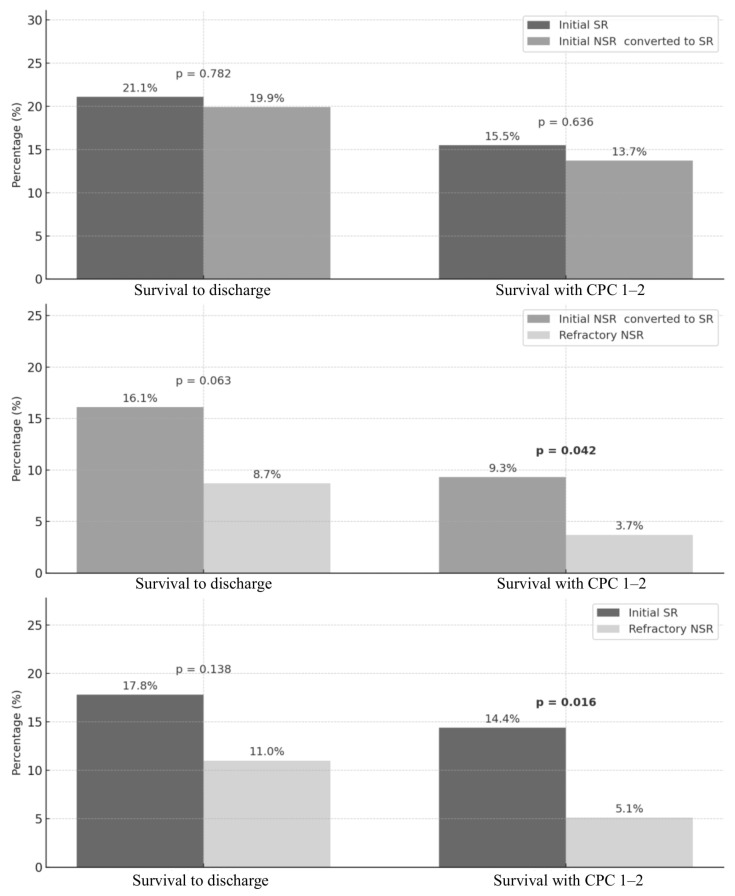
Outcomes of patients according to cardiac rhythm after propensity score matching. SR, shockable rhythm; NSR, non-shockable rhythm; CPC, cerebral performance.

- Initial SR vs. initial NSR converted to SR group (n = 322):
  Survival to hospital discharge was slightly higher in the initial SR group compared to the converted group (21.1% vs. 19.9%), but the difference was not statistically significant (*p* = 0.782). Similarly, a favorable neurological outcome (CPC 1–2) was observed in 15.5% of the initial SR group vs. 13.7% of the converted group (*p* = 0.636) (Table 2 and Figure 2).
- Initial NSR converted to SR group vs. refractory NSR group (n = 322):
  Survival to hospital discharge was higher in the initial NSR converted to SR group (16.1%) than in the refractory NSR group (8.7%), but the difference was not statistically significant (*p* = 0.063). However, favorable neurological outcomes occurred more frequently in the initial NSR converted to SR group (9.3%) than in the initial NSR group (3.7%), with a statistically significant difference (*p* = 0.042) (Table 3 and Figure 2).
- Initial SR vs. refractory NSR group (n = 236):
  Survival to discharge was higher in the initial SR group (17.8%) compared to the refractory NSR group (11.0%), though the difference was not statistically significant (*p* = 0.138). However, the initial SR group had a significantly better neurological outcome (CPC 1–2) than the refractory NSR group (14.4% vs. 5.1%, *p* = 0.016) (Table 4 and Figure 2).

### 3.4. Multivariable Logistic Analysis of Outcomes in the Patient Groups After Propensity Score Matching

No statistically significant differences were observed in AOR for survival to hospital discharge among the three comparison groups. However, for favorable neurological outcomes (CPC 1–2), patients in the refractory NSR group had significantly lower odds compared to the other groups.

**Figure 3:**
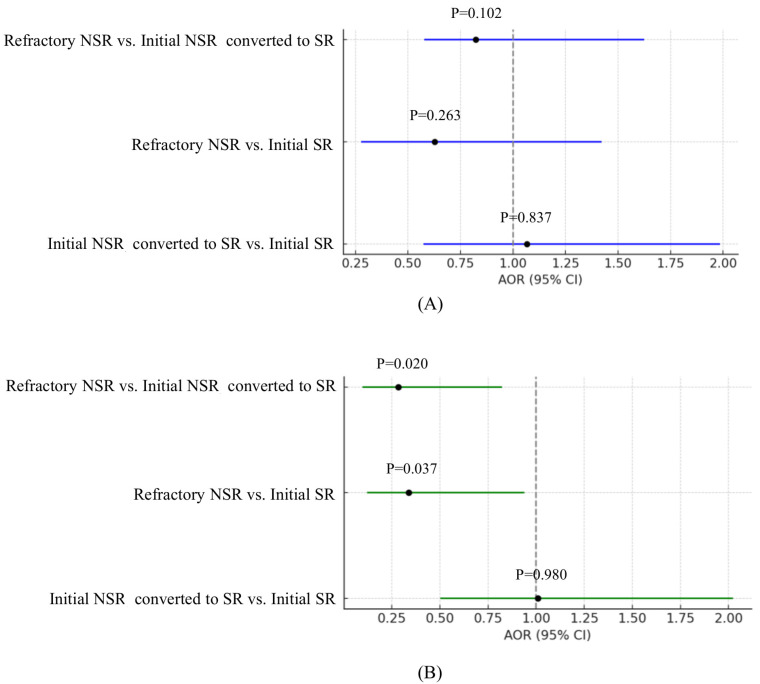
Outcomes of multivariable logistic regression analysis for outcomes of patients after propensity score matching. (**A**) Survival to hospital discharge; (**B**) survival with CPC 1–2. Abbreviations: SR, shockable rhythm; NSR, non-shockable rhythm; AOR, adjusted odds ratio; CI, confidence interval.

- Compared with the initial SR group, the refractory NSR group had an AOR of 0.337 (95% CI: 0.121–0.937; *p* = 0.037).
- Compared with the initial NSR converted to SR group, the refractory NSR group had an AOR of 0.283 (95% CI: 0.097–0.822; *p* = 0.020) (Table 5 and Figure 3).

## 4. Discussion

This study highlights the prognostic significance of initial cardiac rhythm and rhythm conversion prior to ECPR initiation in patients with refractory OHCA. Patients presenting with an initial SR had the most favorable neurological outcomes, as measured by survival with CPC 1–2, followed by those whose NSR converted to SR before ECPR initiation. In contrast, patients with a persistent NSR exhibited the poorest neurological outcomes.

These findings are consistent with previous studies showing that ECPR offers survival benefits in patients with OHCA with an initial SR, particularly in the context of bystander CPR and rapid transport to hospital care [12]. Moreover, other studies have reported that patients with an initial NSR who subsequently converted to SR had significantly better outcomes than those with persistent NSR [13,14]. These results support the hypothesis that rhythm conversion may serve as a surrogate marker of physiological viability and recovery potential and could help identify patients who are more likely to benefit from ECPR.

Our propensity score-matched analysis reinforces this prognostic gradient. Survival with CPC 1–2 was significantly higher in the initial SR group compared to the persistent NSR group (14.4% vs. 5.1%, *p* = 0.016). Among patients initially presenting with NSR, those who achieved rhythm conversion had better neurological outcomes than those who did not (9.3% vs. 3.7%, *p* = 0.042). These findings suggest that rhythm conversion before ECPR cannulation is not only a prognostic indicator but could also be a potential therapeutic target during resuscitation.

The recent literature supports these observations. For instance, Rob et al. found that patients presenting with and maintaining ventricular fibrillation had higher rates of favorable neurological outcomes. In contrast, those with asystole or conversion to asystole had poor outcomes [10]. These results align with earlier OHCA studies in non-ECPR populations, which identified conversion to a shockable rhythm as an indicator of better prognosis [10,11].

Furthermore, a recent single-center study utilizing a machine learning model identified cardiac rhythm status at the time of ECMO cannulation as the most predictive variable for favorable neurological outcomes following ECPR [15]. Although their cohort included only patients with initial VF, the predictive value of rhythm status corroborates our findings. Collectively, these results underscore the importance of rhythm assessment in the selection of ECPR candidates and early prognostication.

From a physiological standpoint, rhythm conversion may reflect preserved myocardial viability and the presence of a potentially reversible phase of cardiac arrest. Timely resuscitation and extracorporeal support during this window can facilitate organ recovery. Conversely, persistent NSR typically indicates a prolonged no-flow or low-flow state, leading to hypoperfusion and progression to a metabolic phase associated with irreversible damage to the brain and heart [8,10,11,16]. In such cases, the potential benefit of ECPR is significantly reduced.

Notably, our study also identified differences in post-arrest care based on rhythm status. Patients with initial SR or conversion to SR were more likely to receive advanced post-resuscitation interventions, such as coronary reperfusion and TTM, compared to those with persistent NSR. This disparity may reflect a clinical inclination to administer more aggressive post-arrest therapies in patients perceived to have a more favorable prognosis based on their rhythm status.

### Limitations

This study had certain limitations that warrant consideration. First, its retrospective design inherently limits causal inference and introduces selection and reporting biases. Although propensity score matching was employed to reduce confounding, the influence of unmeasured variables cannot be entirely excluded. Second, variability in ECPR protocols, resuscitation approaches, and postcardiac arrest care across participating institutions may have influenced patient outcomes and limited the generalizability of the findings. Third, while survival with favorable neurological outcomes was evaluated, detailed assessments of long-term neurological function and health-related quality of life were not available. Finally, precise documentation of time intervals, such as the no-flow time (i.e., the interval from collapse to initiation of CPR), was not documented. Given the known influence of no-flow duration on neurological outcomes, this missing information may have confounded the observed association between rhythm dynamics and prognosis.

Despite these limitations, the study provides clinically relevant insights into the prognostic value of rhythm dynamics before ECPR. Future prospective studies with standardized protocols, comprehensive long-term follow-up, and accurate documentation of resuscitation timelines are needed to validate and extend these findings.

## 5. Conclusions

Rhythm conversion to SR during CPR in patients with an initial NSR may serve as an important prognostic marker and potential resuscitation target. These findings support the implementation of tailored resuscitation strategies aimed at achieving rhythm conversion prior to ECPR initiation. Incorporating rhythm dynamics into clinical decision-making may enhance patient selection for ECPR and optimize outcomes.

## Figures and Tables

**Table 1:** Characteristics of the study population according to initial and converted cardiac rhythm before ECPR initiation.

Variables	Total	Initial SR	Initial NSR Converted to SR	Refractory NSR	*p*
	(N = 681)	(N = 161)	(N = 345)	(N = 175)	
Sex, male, n (%)	570 (83.7%)	139 (86.3%)	301 (87.2%)	130 (74.3%)	<0.001
Age, years [median (IQR)]	56 [46–64]	54 [46–63]	55 [45–62]	60 [47–68]	0.004
Bystander CPR	453 (66.5%)	99 (61.5%)	239 (69.3%)	115 (65.7%)	0.217
Witnessed arrest	563 (82.7%)	133 (82.6%)	288 (83.5%)	142 (81.1%)	0.801
Cardiac origin	638 (93.7%)	154 (95.7%)	328 (95.1%)	156 (89.1%)	0.016
Pre-existing comorbidity, n (%)					
HTN	249 (36.6%)	53 (32.9%)	134 (38.8%)	62 (35.4%)	0.409
DM	165 (24.2%)	35 (21.7%)	82 (23.8%)	48 (27.4%)	0.459
Heart disease	135 (19.8%)	33 (20.5%)	68 (19.7%)	34 (19.4%)	0.968
Respiratory disease	19 (2.8%)	6 (3.7%)	12 (3.5%)	1 (0.6%)	0.091
CKD	20 (2.9%)	3 (1.9%)	9 (2.6%)	8 (4.6%)	0.345
Stroke	23 (3.4%)	5 (3.1%)	9 (2.6%)	9 (5.1%)	0.312
Mechanical CPR	223 (32.7%)	40 (24.8%)	121 (35.1%)	62 (35.4%)	0.456
Post-cardiac arrest care					
Reperfusion treatment ^a^	447 (65.6%)	116 (72.0%)	241 (69.9%)	90 (51.4%)	<0.001
TTM	114 (16.7%)	33 (20.5%)	67 (19.4%)	14 (8.0%)	0.002
Transient pre-ECPR ROSC ^b^	102 (15.0%)	23 (14.3%)	52 (15.1%)	27 (15.4%)	0.956
Time interval, mins					
Call or witness to ED *	42.0 [23.0–74.0]	65.5 [25.0–74.0]	38.0 [22.0–72.5]	40.0 [22.0–75.5]	0.182
From ED to ECPR ^$^	80.5 [43.0–99.0]	75.0 [35.0–97.0]	82.0 [52.5–99.0]	82.0 [49.0–102.5]	0.045
Outcome at hospital discharge					
Survival to discharge	118 (17.3%)	34 (21.1%)	67 (19.4%)	17 (9.7%)	0.008
Survival with CPC 1–2	72 (10.6%)	25 (15.5%)	40 (11.6%)	7 (4.0%)	0.002

Abbreviations: IQR, interquartile range; CPR, cardiopulmonary resuscitation; DM, diabetes mellitus; HTN, hypertension; CKD, chronic kidney disease; TTM, targeted temperature management; ECPR, extracorporeal cardiopulmonary resuscitation; ROSC, return of spontaneous circulation; TI, time interval; ER, emergency room. * Time from EMS call or witnessed arrest to ED arrival; ^$^ Time from ED arrival to ECMO pump-on at ED. ^a^ Reperfusion treatment was defined as intravenous thrombolysis and percutaneous coronary intervention. ^b^ Transient pre-ECPR ROSC (duration > 1 min and <20 min) prior to ECPR initiation. Male patients predominated across all groups (overall 83.7%), with significantly higher proportions in the initial SR (86.3%) and converted (87.2%) groups compared to the refractory NSR group (74.3%, *p* < 0.001). The refractory NSR group had a significantly higher median age (60 years; IQR, 47–68) than both the initial SR (54 years; IQR, 46–63) and converted groups (55 years; IQR, 45–62) (*p* = 0.004). Cardiac etiologies of arrest were more frequently reported in the initial SR and converted groups than in the refractory NSR group (*p* = 0.016). Reperfusion therapy (e.g., thrombolysis or PCI) and TTM were significantly more common in the initial SR and converted groups (*p* < 0.001 and *p* = 0.002, respectively). Furthermore, the median time from ED arrival to ECPR initiation was shortest in the initial SR group (*p* = 0.045). No significant intergroup differences were observed in the rates of bystander CPR, witnessed arrest, presence of preexisting comorbidities, or prehospital time intervals.

**Table 2:** Characteristics of the study population according to cardiac rhythm after propensity score matching between the initial SR group and initial NSR converted to SR group.

Variables	Total	Initial SR	Initial NSR Converted to SR	*p*
	(N = 322)	(N = 161)	(N = 161)	
Sex, male, n (%)	271 (84.2%)	139 (86.3%)	132 (82.0%)	0.285
Age, years (IQR)	54.0 [44.0–63.0]	54.0 [46.0–63.0]	54.0 [42.0–62.0]	0.469
Bystander CPR	200 (62.1%)	99 (61.5%)	101 (62.7%)	0.818
Witnessed arrest	268 (83.2%)	133 (82.6%)	135 (83.9%)	0.765
Cardiac origin	306 (95.0%)	154 (95.7%)	152 (94.4%)	0.608
Pre-existing comorbidity, n (%)				
HTN	114 (35.4%)	53 (32.9%)	61 (37.9%)	0.351
DM	79 (24.5%)	35 (21.7%)	44 (27.3%)	0.244
Heart disease	61 (18.9%)	33 (20.5%)	28 (17.4%)	0.477
Respiratory disease	9 (2.8%)	6 (3.7%)	3 (1.9%)	0.310
CKD	6 (1.9%)	3 (1.9%)	3 (1.9%)	1.000
Stroke	8 (2.5%)	5 (3.1%)	3 (1.9%)	0.474
Mechanical CPR	90 (28.0%)	40 (24.8%)	50 (31.1%)	0.214
Post-cardiac arrest care				
Reperfusion treatment ^a^	228 (70.8%)	116 (72.0%)	112 (69.6%)	0.624
TTM	72 (22.4%)	33 (20.5%)	39 (24.2%)	0.422
Transient Pre-ECPR ROSC ^b^	49 (15.2%)	23 (14.3%)	26 (16.1%)	0.642
Time interval, mins				
Call or witness to ED *	57.0 [24.0–74.0]	65.5 [25.0–74.0]	42.0 [23.0–73.0]	0.153
From ED to ECPR ^$^	78.0 [38.0–99.0]	75.0 [35.0–97.0]	81.0 [48.0–101.0]	0.054
Outcome at hospital discharge				
Survival to discharge	66 (20.5%)	34 (21.1%)	32 (19.9%)	0.782
Survival with CPC 1–2	47 (14.6%)	25 (15.5%)	22 (13.7%)	0.636

Abbreviations: SR, shockable rhythm; NSR, non-shockable rhythm; IQR, interquartile range; CPR, cardiopulmonary resuscitation; DM, diabetes mellitus; HTN, hypertension; CKD, chronic kidney disease; TTM, targeted temperature management; ECPR, extracorporeal cardiopulmonary resuscitation; ROSC, return of spontaneous circulation; ED, emergency department. * Time from EMS call or witnessed arrest to ED arrival. ^$^ Time from ED arrival to ECMO pump-on at the ED. ^a^ Reperfusion treatment was defined as intravenous thrombolysis and percutaneous coronary intervention. ^b^ Transient pre-ECPR ROSC (duration > 1 min and <20 min) prior to ECPR initiation.

**Table 3:** Characteristics of the study population according to cardiac rhythm after propensity score matching between the initial NSR converted to SR group and the refractory NSR group.

Variables	Total	Initial NSR Converted to SR	Refractory NSR	*p*
	(N = 322)	(N = 161)	(N = 161)	
Sex, male, n (%)	254 (78.9%)	125 (77.6%)	129 (80.1%)	0.585
Age, years (IQR)	58.0 [47.0–65.0]	58.0 [48.0–64.0]	59.0 [46.0–67.0]	0.411
Bystander CPR	214 (66.5%)	107 (66.5%)	107 (66.5%)	1.000
Witnessed arrest	262 (81.4%)	131 (81.4%)	131 (81.4%)	1.000
Cardiac origin	298 (92.5%)	149 (92.5%)	149 (92.5%)	1.000
Pre-existing comorbidity, n (%)				
HTN	111 (34.5%)	53 (32.9%)	58 (36.0%)	0.558
DM	80 (24.8%)	37 (23.0%)	43 (26.7%)	0.439
Heart disease	64 (19.9%)	31 (19.3%)	33 (20.5%)	0.780
Respiratory disease	3 (0.9%)	2 (1.2%)	1 (0.6%)	0.562
CKD	13 (4.0%)	5 (3.1%)	8 (5.0%)	0.396
Stroke	18 (5.6%)	9 (5.6%)	9 (5.6%)	1.000
Mechanical CPR	117 (36.3%)	60 (37.3%)	57 (35.4%)	0.728
Post-cardiac arrest care				
Reperfusion treatment ^a^	178 (55.3%)	89 (55.3%)	89 (55.3%)	1.000
TTM	29 (9.0%)	15 (9.3%)	14 (8.7%)	0.846
Transient Pre-ECPR ROSC ^b^	42 (13.0%)	18 (11.2%)	24 (14.9%)	0.321
Time interval, mins				
Call or witness to ED *	36.0 [20.0–73.0]	30.0 [18.0–68.0]	40.0 [24.0–76.0]	0.010
From ED to ECPR ^$^	81.0 [49.5–101.0]	80.0 [55.0–99.0]	81.5 [49.0–102.0]	0.932
Outcome at hospital discharge				
Survival to discharge	40 (12.4%)	26 (16.1%)	14 (8.7%)	0.063
Survival with CPC1–2	21 (6.5%)	15 (9.3%)	6 (3.7%)	0.042

Abbreviations: SR, shockable rhythm; NSR, non-shockable rhythm; IQR, interquartile range; CPR, cardiopulmonary resuscitation; DM, diabetes mellitus; HTN, hypertension; CKD, chronic kidney disease; TTM, targeted temperature management; ECPR, extracorporeal cardiopulmonary resuscitation; ROSC, return of spontaneous circulation; ED, emergency department. * Time from EMS call or witnessed arrest to ED arrival. ^$^ Time from ED arrival to ECMO pump-on at the ED. ^a^ Reperfusion treatment was defined as intravenous thrombolysis and percutaneous coronary intervention. ^b^ Transient pre-ECPR ROSC (duration > 1 min and <20 min) prior to ECPR initiation.

**Table 4:** Characteristics of the study population according to cardiac rhythm after propensity score matching between the initial SR group and the refractory NSR group.

Variables	Total	Initial SR	Refractory NSR	*p*
	(N = 236)	(N = 118)	(N = 118)	
Sex, male, n (%)	193 (81.8%)	97 (82.2%)	96 (81.4%)	0.866
Age, years (IQR)	58.0 [47.0–65.5]	57.5 [48.0–65.0]	58.5 [45.0–66.0]	0.579
Bystander CPR	149 (63.1%)	74 (62.7%)	75 (63.6%)	0.893
Witnessed arrest	194 (82.2%)	96 (81.4%)	98 (83.1%)	0.734
Cardiac origin	224 (94.9%)	113 (95.8%)	111 (94.1%)	0.553
Pre-existing comorbidity, n (%)				
HTN	79 (33.5%)	36 (30.5%)	43 (36.4%)	0.334
DM	59 (25.0%)	30 (25.4%)	29 (24.6%)	0.881
Heart disease	51 (21.6%)	24 (20.3%)	27 (22.9%)	0.635
Respiratory disease	2 (0.8%)	1 (0.8%)	1 (0.8%)	1.000
CKD	7 (3.0%)	3 (2.5%)	4 (3.4%)	0.701
Stroke	9 (3.8%)	5 (4.2%)	4 (3.4%)	0.734
Mechanical CPR	66 (28.0%)	33 (28.0%)	33 (28.0%)	1.000
Post-cardiac arrest care				
Reperfusion treatment ^a^	146 (61.9%)	76 (64.4%)	70 (59.3%)	0.421
TTM	29 (12.3%)	17 (14.4%)	12 (10.2%)	0.321
Transient pre-ECPR ROSC ^b^	32 (13.6%)	16 (13.6%)	16 (13.6%)	1.000
Time interval, mins				
Call or witness to ED *	57.0 [24.0–76.0]	65.5 [27.0–74.0]	40.0 [21.0–78.0]	0.749
From ED to ECPR ^$^	77.0 [37.0–103.0]	73.0 [35.5–101.5]	79.0 [46.0–106.0]	0.102
Outcome at hospital discharge				
Survival to discharge	34 (14.4%)	21 (17.8%)	13 (11.0%)	0.138
Survival with CPC1–2	23 (9.7%)	17 (14.4%)	6 (5.1%)	0.016

Abbreviations: SR, shockable rhythm; NSR, non-shockable rhythm; IQR, interquartile range; CPR, cardiopulmonary resuscitation; DM, diabetes mellitus; HTN, hypertension; CKD, chronic kidney disease; TTM, targeted temperature management; ECPR, extracorporeal cardiopulmonary resuscitation; ROSC, return of spontaneous circulation; ED, emergency department. * Time from EMS call or witnessed arrest to ED arrival. ^$^ Time from ED arrival to ECMO pump-on at the ED. ^a^ Reperfusion treatment was defined as intravenous thrombolysis and percutaneous coronary intervention. ^b^ Transient pre-ECPR ROSC (duration > 1 min and <20 min) prior to ECPR initiation.

**Table 5:** Multivariable logistic regression analysis for outcomes of patients between three groups after the propensity score matching.

Outcomes	Groups	Initial SR (Ref.) vs. Initial NSR Converted to SR (Test)			Initial SR (Ref.) vs. Refractory NSR (Test)			Initial NSR Converted to SR (Ref.) vs. Refractory NSR (Test)		
		AOR	95% CI	*p*	AOR	95% CI	*p*	AOR	95% CI	*p*
Survival to discharge *	Reference	1.00			1.00			1.00		
	Test	1.067	0.574–1.985	0.837	0.627	0.277–1.421	0.263	0.823	0.577–1.624	0.102
Survival with CPC 1–2 *	Reference	1.00			1.00			1.00		
	Test	1.009	0.503–2.023	0.980	0.337	0.121–0.938	0.037	0.283	0.097–0.822	0.020

Abbreviations: SR, shockable rhythm; NSR, non-shockable rhythm; AOR, adjusted odds ratio; CI, confidence interval. * Adjusted odds ratio for sex, age, bystander CPR, witnessed arrest, cardiac origin, preexisting comorbidity, mechanical CPR, reperfusion treatment, targeted temperature management, transient pre-ECPR ROSC, time interval from call or witnessed arrest to ER arrival, and time interval from ER arrival to ECPR.

## Data Availability

The authors used a database made available by the Korea Disease Control and Prevention Agency, which holds authority over the OHCA registry dataset in Korea. Access to this dataset requires permission. Interested parties can request access through the official website (https://www.kdca.go.kr/injury/biz/injury/main/mainPage.do) (accessed on 25 March 2025).

## Supplementary Materials

The following supporting information can be downloaded at: https://www.mdpi.com/article/10.3390/jcm14145066/s1, Figure S1: Changes in absolute standardized mean differences (**A**) and dot plot of absolute standardized mean differences (**B**) in ECPR patients with an initial SR and those with an initial NSR converted to SR, before and after propensity score matching; Figure S2: Changes in absolute standardized mean differences (**A**) and dot plot of absolute standardized mean differences (**B**) in ECPR patients with an initial NSR converted to SR and those with a refractory NSR, before and after propensity score matching; Figure S3: Changes in absolute standardized mean differences (**A**) and dot plot of absolute standardized mean differences (**B**) in ECPR patients with an initial SR and those with a refractory NSR, before and after propensity score matching; Table S1: Definition and detailed classification of pre-existing comorbidity; Table S2: Glasgow–Pittsburgh Cerebral Performance Category (CPC) scores and their corresponding descriptions.

## Abbreviations

The following abbreviations are used in this manuscript:

AOR	Adjusted Odds Ratio
CPC	Cerebral Performance Category
CPR	Cardiopulmonary Resuscitation
ECPR	Extracorporeal Cardiopulmonary Resuscitation
ECMO	Extracorporeal Membrane Oxygenation
NSR	Non-Shockable Rhythm
OHCA	Out-of-Hospital Cardiac Arrest
OHCAS	Out-of-Hospital Cardiac Arrest Surveillance
SR	Shockable Rhythm
TTM	Targeted Temperature Management
VF	Ventricular Fibrillation
