# Supplementary material for: Clinical Significance of Initial and Converted Cardiac Rhythms in Extracorporeal Cardiopulmonary Resuscitation for Patients with Refractory Out-of-Hospital Cardiac Arrest: A Nationwide Observational Study
Source: J Clin Med. 2025 Jul 17;14(14):5066. doi: 10.3390/jcm14145066 (PMC12296152; doi:10.3390/jcm14145066)
Supplement: Supplementary file 1 [file jcm-14-05066-s001.zip › jcm-3733778-supplementary.pdf]

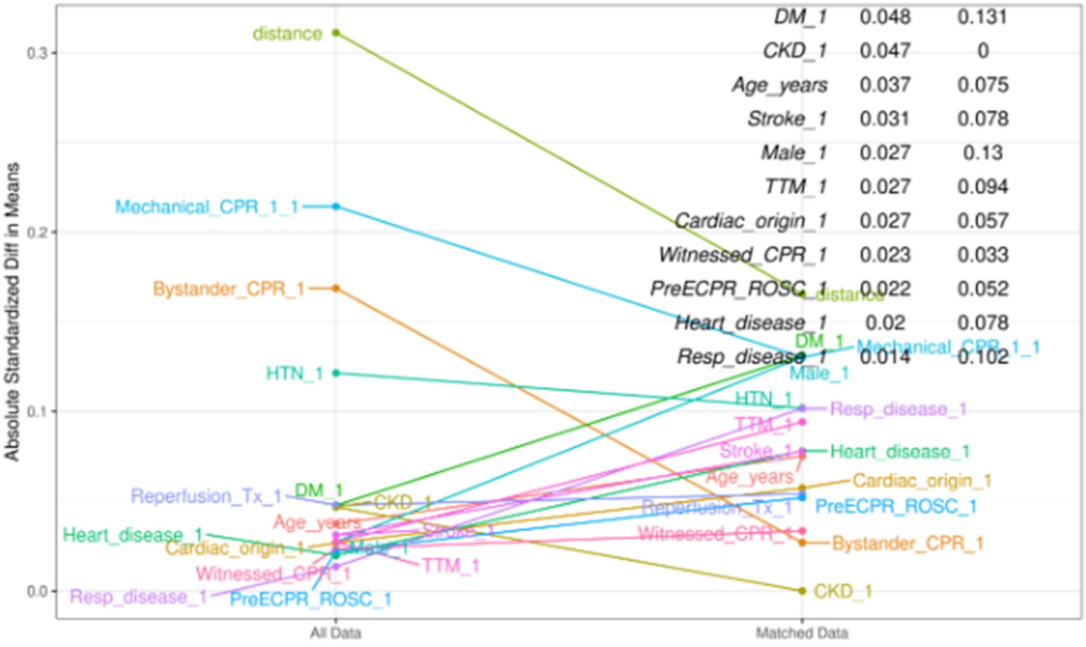

(A)

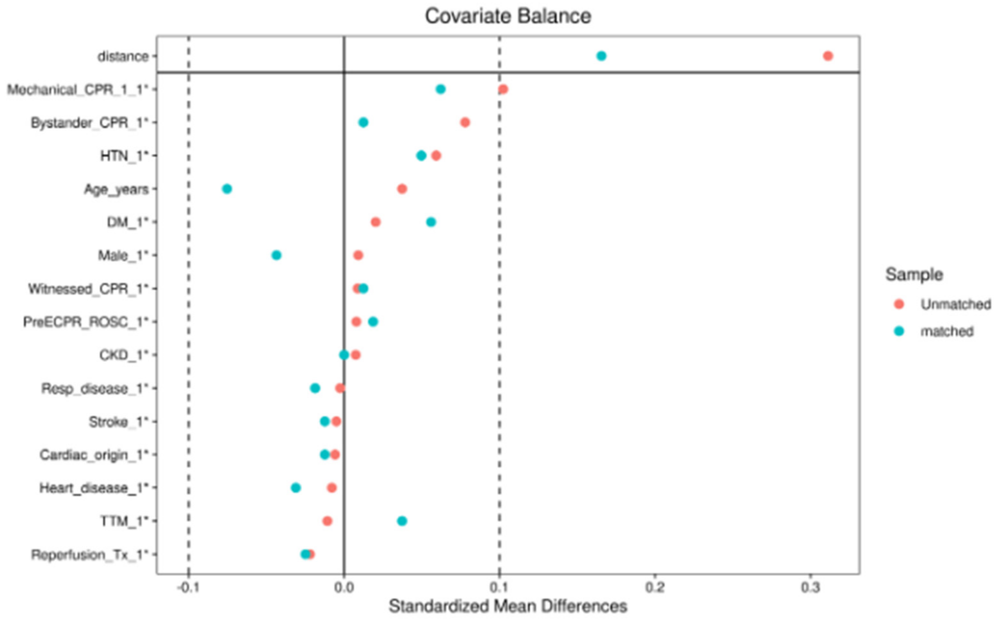

(B)

Figure S1. Changes in absolute standardized mean differences (A) and dot plot of absolute standardized mean differences (B) in ECPR patients with an initial SR and those with an initial NSR converted to SR, before and after propensity score matching.

Abbreviations: CA, cardiac arrest; CPR, cardiopulmonary resuscitation; CKD, chronic kidney disease; DM, diabetes mellitus; HTN, hypertension; ROSC, return of spontaneous circulation; ER, emergency room, TTM, targeted temperature management; ECPR, extracorporeal cardiopulmonary resuscitation; EMS, emergency medical services; NSR, non-shockable rhythm; SR, shockable rhythm.

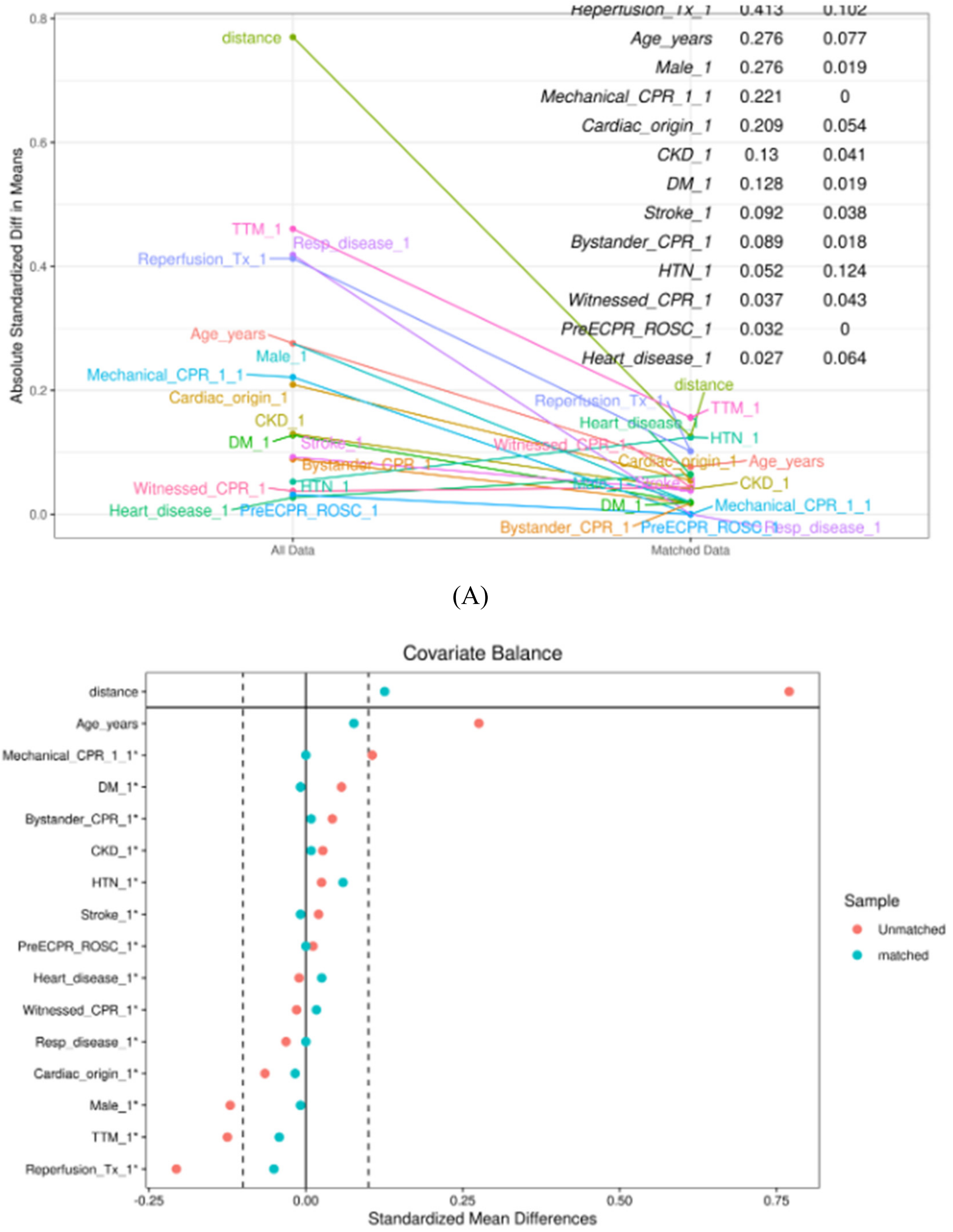

(A)

(B)

Figure S2. Changes in absolute standardized mean differences (A) and dot plot of absolute standardized mean differences (B) in ECPR patients with an initial NSR converted to SR and those with a refractory NSR, before and after propensity score matching.

Abbreviations: CA, cardiac arrest; CPR, cardiopulmonary resuscitation; CKD, chronic kidney disease; DM, diabetes mellitus; HTN, hypertension; ROSC, return of spontaneous circulation; ER, emergency room, TTM, targeted temperature management; ECPR, extracorporeal cardiopulmonary resuscitation; EMS, emergency medical services; NSR, non-shockable rhythm; SR, shockable rhythm.

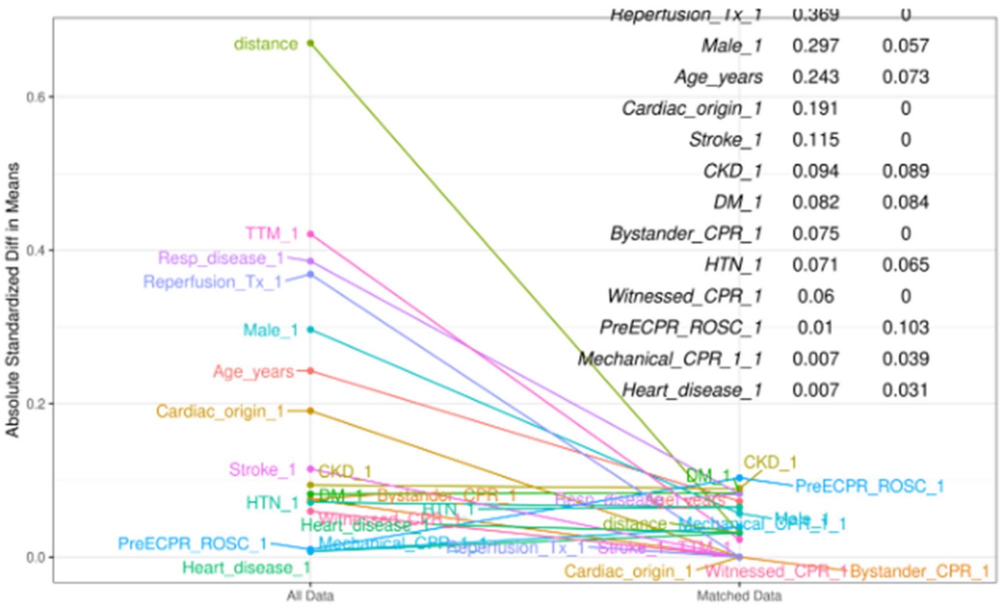

(A)

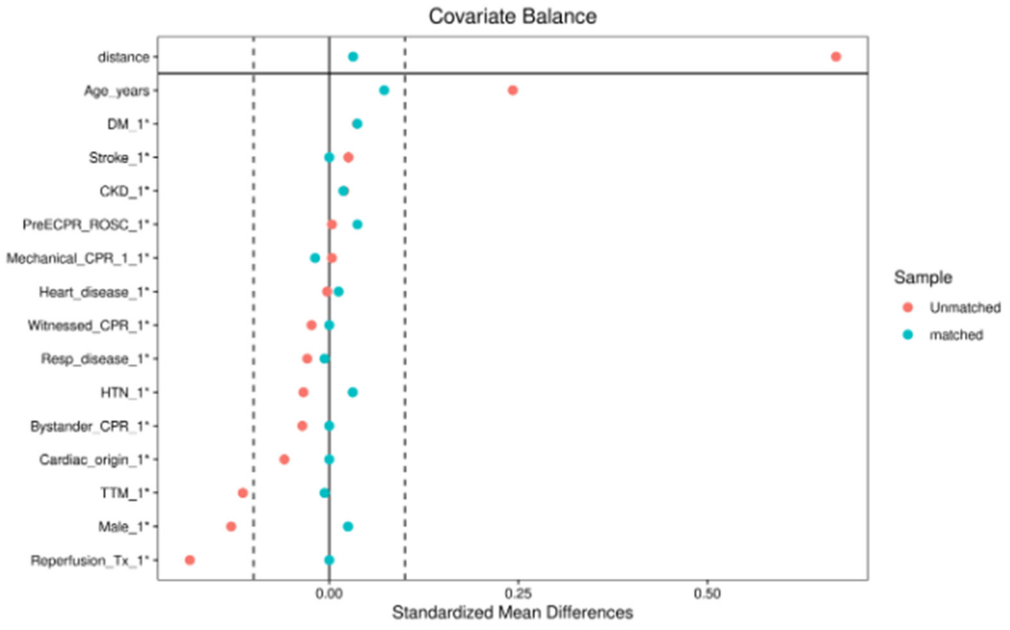

(B)

Figure S3. Changes in absolute standardized mean differences (A) and dot plot of absolute standardized mean differences (B) in ECPR patients with an initial SR and those with a refractory NSR, before and after propensity score matching.

Abbreviations: CA, cardiac arrest; CPR, cardiopulmonary resuscitation; CKD, chronic kidney disease; DM, diabetes mellitus; HTN, hypertension; ROSC, return of spontaneous circulation; ER, emergency room, TTM, targeted temperature management; ECPR, extracorporeal cardiopulmonary resuscitation; EMS, emergency medical services; NSR, non-shockable rhythm; SR, shockable rhythm.

Supplementary Table 1. Definition and detailed classification of pre-existing comorbidity

Disease	Definition and detailed classification
Hypertension	The disease was diagnosed by the doctor prior to cardiac arrest and is clearly stated in the medical record
Diabetes mellitus	The disease was diagnosed by the doctor prior to cardiac arrest and is clearly stated in the medical record.
Heart disease	The disease was diagnosed by the doctor prior to cardiac arrest and is clearly stated in the medical record. (Ischemic heart disease, myocardial infarction, angina, valvular heart disease, arrhythmia, congestive heart disease, myocardiopathy, congenital heart disease, previous heart intervention record including stent, primary coronary intervention, coronary artery bypass grafting, and other heart disease)
Chronic renal disease	The disease was diagnosed by the doctor prior to cardiac arrest and is clearly stated in the medical record. (chronic renal failure, dialysis, kidney transplantation, and other chronic kidney disease)
Chronic respiratory disease	The disease was diagnosed by the doctor prior to cardiac arrest and is clearly stated in the medical record. (Asthma, chronic obstructive pulmonary disease, pulmonary tuberculosis, other chronic respiratory disease.)
Stroke	The disease was diagnosed by the doctor prior to cardiac arrest and is clearly stated in the medical record. (cerebral infarction, cerebral haemorrhage, and unclassified stroke)
Dyslipidemia	The disease was diagnosed by the doctor prior to cardiac arrest and is clearly stated in the medical record. (hyperlipidaemia and unclassified dyslipidemia)

Supplementary Table 2. Glasgow-Pittsburgh Cerebral Performance Categories (CPC) scores and their corresponding descriptions

CPC Score	Description
CPC 1	Good cerebral performance with mild or no neurological deficit
CPC 2	Moderate cerebral disability with moderate neurological deficit, sufficient functional independence for activities of daily living
CPC 3	Severe cerebral disability with severe neurological deficit, dependence on others for daily activities
CPC 4	Coma or vegetative state, absence of meaningful neurological function, inability to interact with the environment
CPC 5	Brain death or death due to neurological injury
